# Supplementary material for: Microscale Thermophoresis Reveals Oxidized Glutathione as High-Affinity Ligand of Mal d 1
Source: Foods. 2021 Nov 11;10(11):2771. doi: 10.3390/foods10112771 (PMC8618550; doi:10.3390/foods10112771)
Supplement: Supplementary file 1 [file foods-10-02771-s001.zip › foods-1429956-supplementary.pdf]

Supplemental material

Microscale Thermophoresis reveals oxidized glutathione as high-affinity ligand of Mal d1

Soraya Chebib[1], Wilfried Schwab[1,*]

[1]Biotechnology of Natural Products, Technical University Munich, Liesel-Beckmann-Str. 1, 85354, Freising, Germany

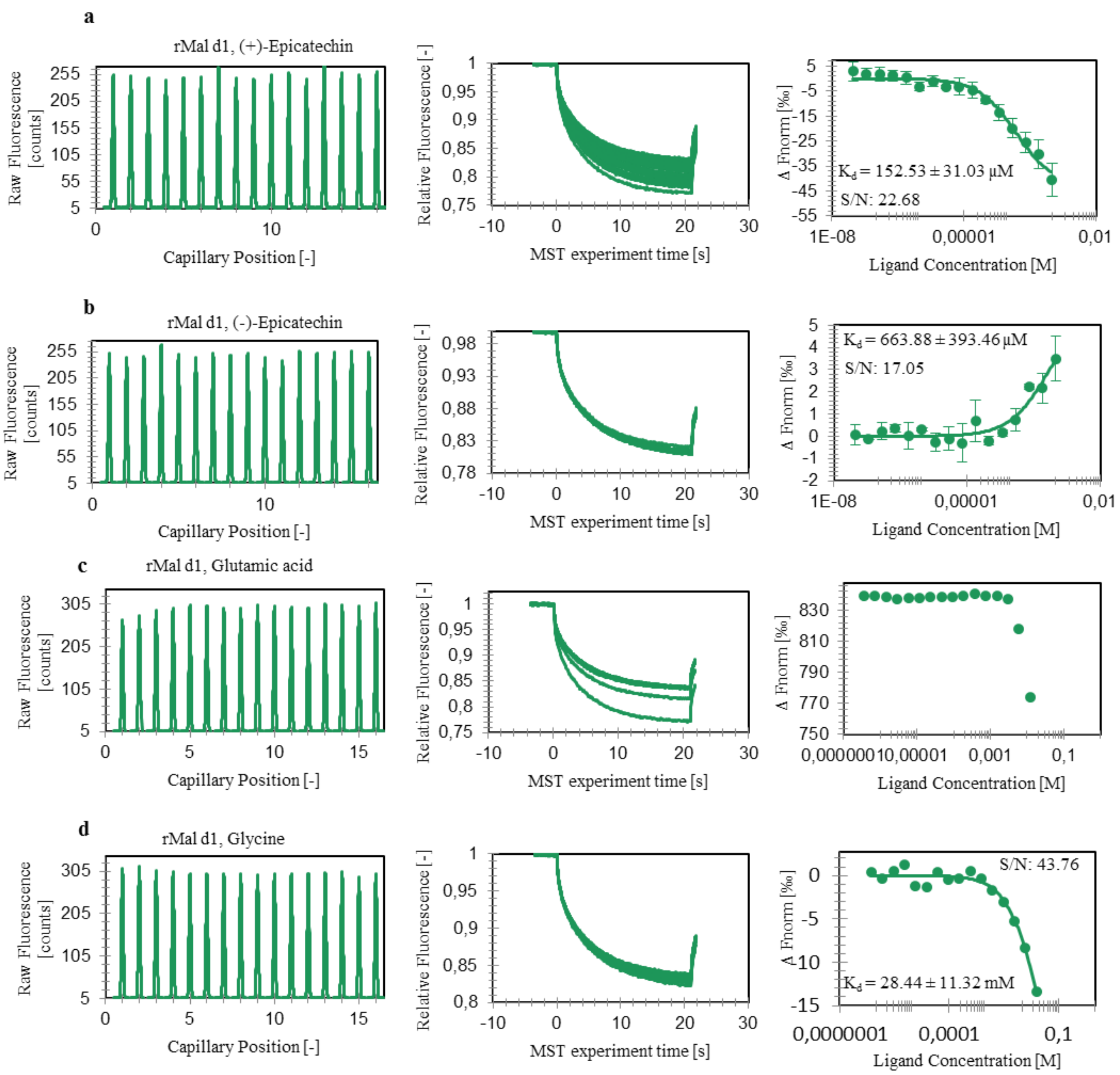

Supplementary Figure S1. Capillary Scan (left), MST traces (middle) and dose-response curves (right) of labeled rMal d1 with (+)-epicatechin (**a**), (-)-epicatechin (**b**), glutamic acid (**c**), and glycine (**d**). Error bars indicate the standard deviation between the performed technical replicates. Dissociation constants (K_d) and signal-to-noise ratios (S/N) are shown.

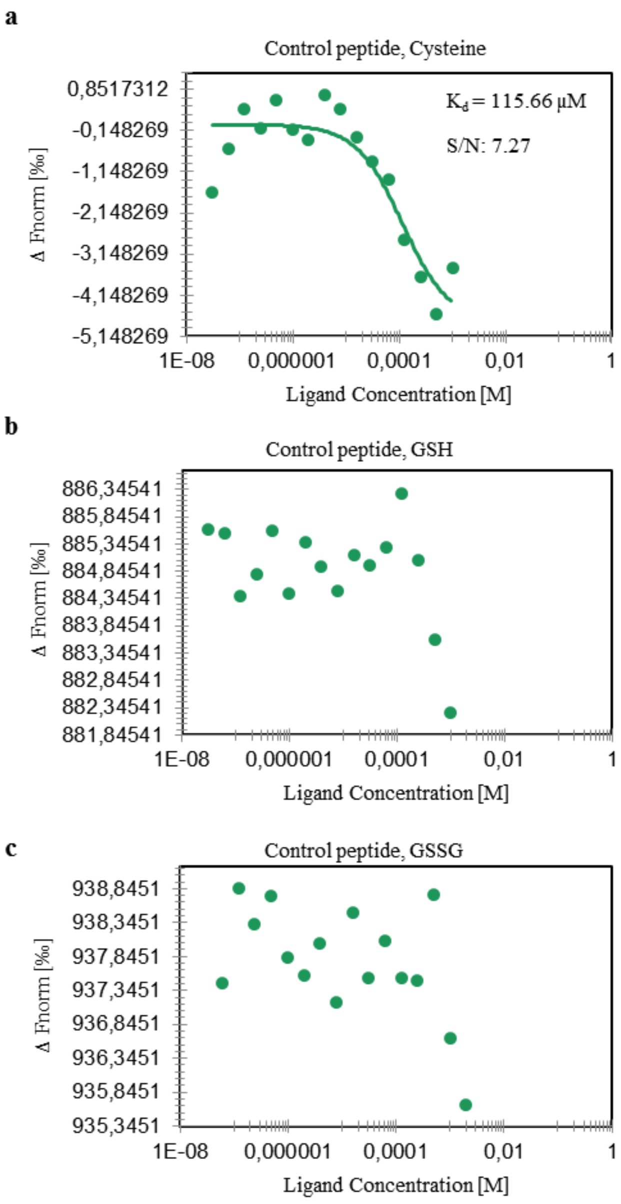

Supplementary Figure S2. Dose-response curves of the control experiments using labeled control peptide and cysteine (**a**), GSH (**b**) and GSSG (**c**).

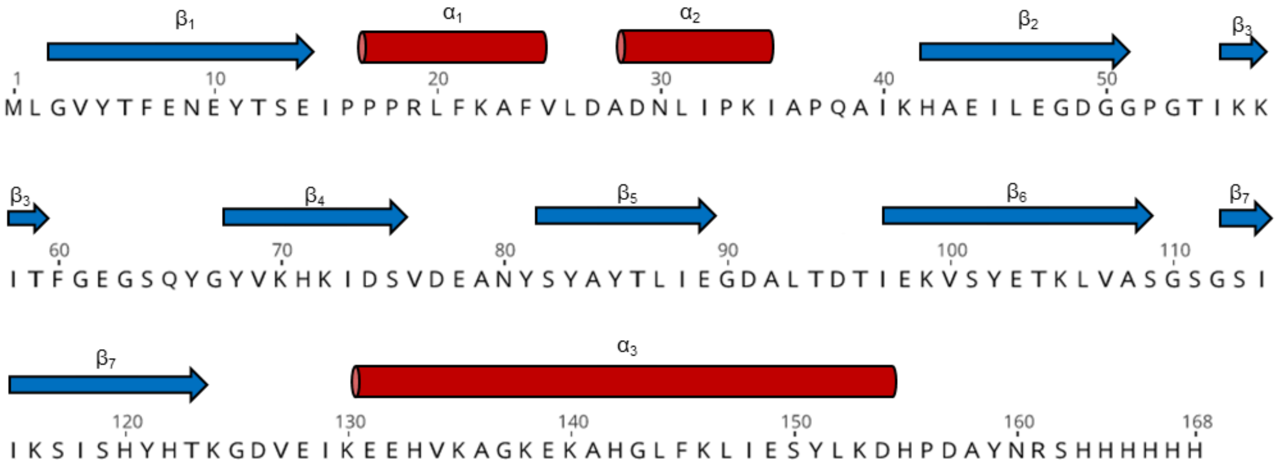

Supplementary Figure S3. Amino acid sequence of recombinant rMal d1.02. Secondary structure elements are shown above the sequence (UniProtKB, https://www.uniprot.org/uniprot/P43211).

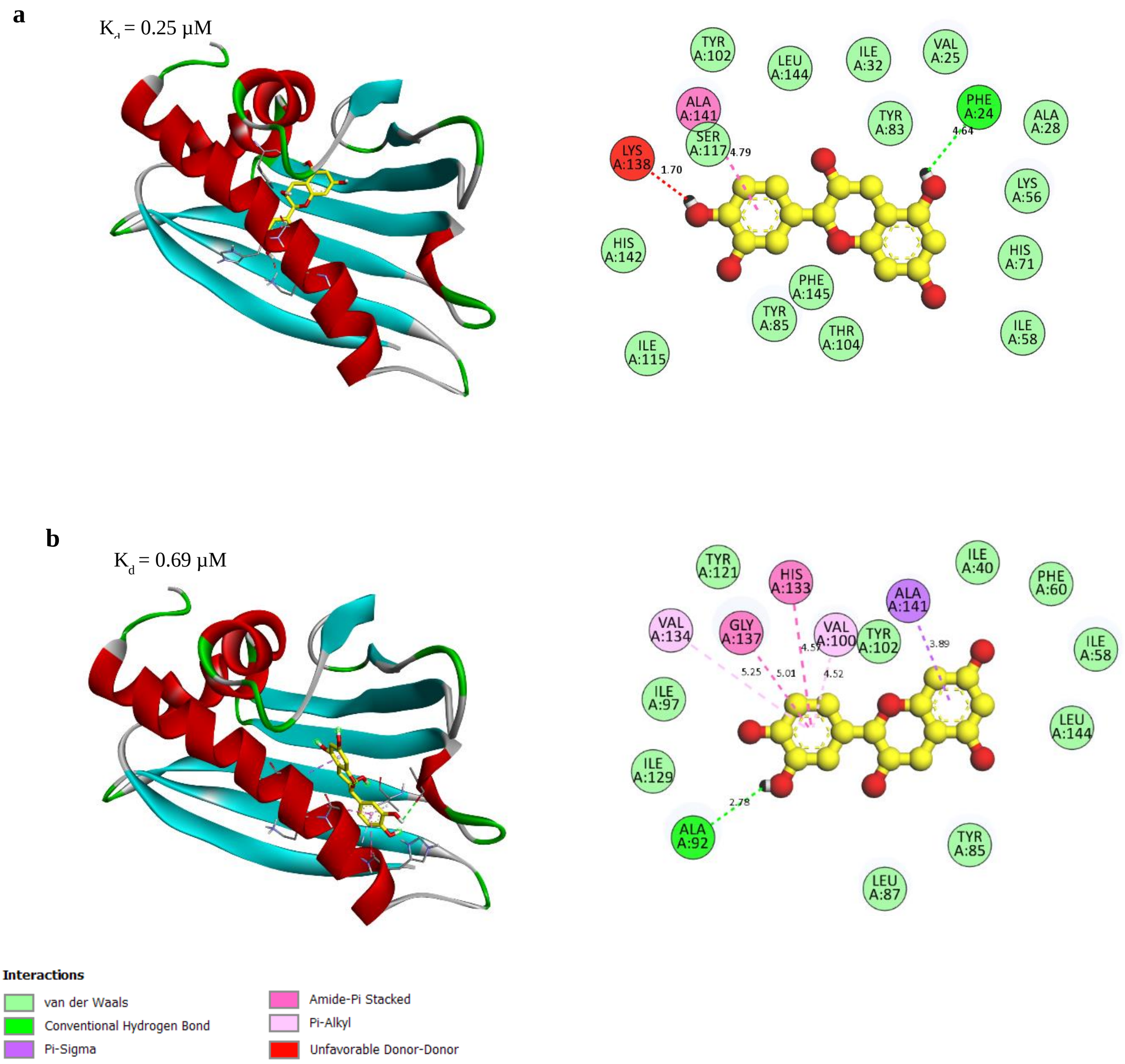

Supplementary Figure S4. 3D- (left) and 2D- (right) interaction model of rMal d1 in complex with (+)-epicatechin (**a**), (-)-epicatechin (**b**).
